# Chloroform in Mania

**Published:** 1849-05

**Authors:** E. B. Moore


*Chloroform in Mania.* By E. B. MOORE, M.D.—Some time since, about 9 o'cl'k P. M., I was called to see a lady, Mrs. H., Laboring under mental alienation. She had been troubled, for some days previous, with *odontalgia*, for which she had applied various "specifics" of her own and friends' recommending. At this time she did not complain of the tooth, but occasionally, she would put her hand to the face, grate her teeth and groan; she would then rise, dance and sing, kick, strike, throw a chair, or anything that came in her way, as she was moved either with pleasure or pain. In short, she was perfectly maniacal. She did not know her own husband or any of her friends. Many efforts were made, without success, to persuade her to have the tooth extracted or to take something to quiet her raving. She said nothing ailed her—that she needed no medicine and would take none; and, as to losing her tooth she had no inclination. It became necessary to keep two or more persons constantly on the watch to prevent her from injuring herself or the things in the room. Under this state of things, I advised her husband to have the chloroform administered as the most certain means of quieting her. It was sent for, and by the assistance of three persons whom it took to hold her, it was administered. She made all the resistance in her power, but in a few minutes she was under its influence. She remained still about fifteen minutes, ween she awoke more rational, and was induced to take an anodyne. In a short time, however, she again became delirious, when the chloroform was administered the second time, and she again became calm. She was now kept under its influence till she was in a sound sleep from the effects of this and the anodyne. She slept quietly from midnight till morning, when she awoke perfectly rational. She then walked half a mile, had her teeth examined, again took the chloroform, and had the defective tooth extracted, without her being conscious when it was done. Since then she has enjoyed a comfortable state of health. I have been in the practice of giving chloroform for the last fifteen months, to alleviate the pains in parturition, in extracting teeth, and for various other affections, and have never yet witnessed any ill effects from its use.—*Boston Med. and Surg. Journal.*

